# AI enabled pulse-echo based SHM system integrated with robotic gripper for the inspection of pipelines

**DOI:** 10.1038/s41598-026-44964-w

**Published:** 2026-04-21

**Authors:** Muhammad Abdullah Tayyab, Hassan Elahi, Muhammad Osama Ali, Anas Bin Aqeel, Ayesha Zeb

**Affiliations:** 1 Department of Mechatronics Engineering, National University of Sciences and Technology PK, Islamabad, Pakistan; 2 Department of Mechanical Engineering, National University of Sciences and Technology PK, Islamabad, Pakistan

**Keywords:** SHM, Pulse-echo, Robotic gripper, Pipeline inspection, Machine learning, Engineering, Materials science

## Abstract

Pipelines are essential infrastructure for moving fluids like water, oil, and gas over long distances. Early and reliable defect detection through non-destructive testing is essential to ensure structural integrity. The main purpose of this work is to propose an automated, AI-enabled pulse-echo ultrasonic structural health monitoring system integrated with a robotic gripper for non-destructive testing of circular pipes. The proposed system features a piezoelectric transducer mounted on a two-claw gripper, which is attached to a 6-DOF collaborative robot (CR5). This setup enables precise and repeatable scanning of steel, aluminum, and silver pipes with varying diameters and wall thicknesses. Experimental results demonstrate the successful excitation and reception of longitudinal guided wave modes, with clear sensitivity to pipe geometry, material properties, crack size (1 cm and 1.5 cm), and crack orientation ($0^\circ$, $45^\circ$, $90^\circ$, and $135^\circ$). Machine learning models, Random Forest, XGBoost, CatBoost, LightGBM, and Logistic Regression, are applied for the classification of defective pipe samples, assessing the percentage loss in the reflected echo’s amplitude, and to determine the crack localization on the surface of circular pipes.

## Introduction

**Fig. 1:**
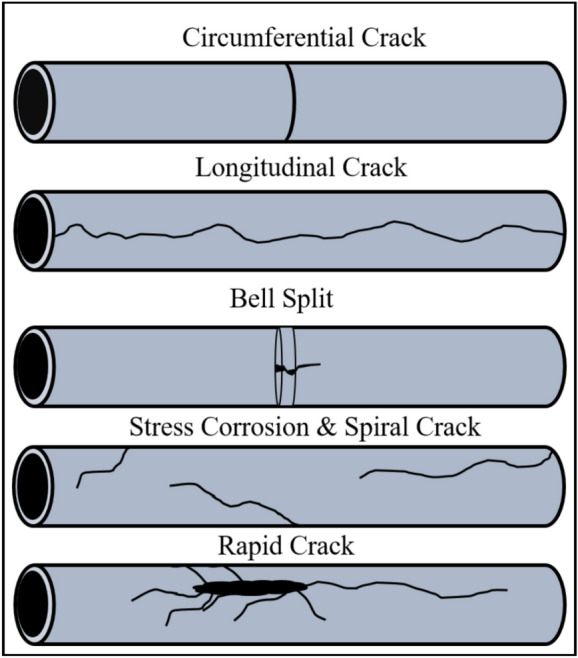
Illustration of failure modes of pipelines.

Structural Health Monitoring (SHM) now become the most emerging and prominent field of engineering science. It involves various kinds of sensors and transducers to assess and manage the condition of structures over time by ensuring the reliability and safety^1,2^. It plays a vital role in the safety of critical structures of buildings, bridges, automobiles, aerospace, etc. A system is said to be damaged when geometric characteristics of material, and boundary conditions change in a way that adversely affects the performance of that system. In general, damage starts at the material level called defects, cracks, and flaws. The propagation of small cracks causes damage to the system^3^. Among the wide range of engineering structures, pipelines, are essential for transporting water, oil, and gas. The most common forms of failure in pipelines are corrosion, stress cracks, seam weld cracks, material flaws, and external damage by excavation equipment. All these common types of pipelines failures are described in the Table 1 and presented in Fig. 1. Thousands of accidents have been reported due to structural failures that are disastrous to both the economy and the environment. Therefore, accurately assessing pipeline structural integrity is critical^4^. For this purpose, condition-based monitoring (CBM) and scheduled-based maintenance (SBM) maintenance and monitoring strategies are adopted to avoid defects and cracks. In SBM whole system is shut down for a short period, and inspection of all tools, equipment, and machines is done in that allocated time. While in CBM different kinds of sensors and transducers are mounted on the surface of the systems’s parts to continuously monitor the performance of the system^5^. SHM is classified into active and passive systems based on the nature of the sensors used, and classified into Destructive Testing (DT) and Non-Destructive Testing (NDT) based on the testing techniques.

**Table 1:** Classification of pipeline’s cracks, their localization, and primary causes^6–8^.

Sr No.	Crack type	Location	Primary cause
1	Longitudinal Crack	Runs parallel to the pipe axis	Internal Pressure, External Loading
2	Circumferential Crack	Runs around the pipe circumference	Axial Tension, Bending Stress
3	Bell Split Crack	Crack at the pipe joint/bell	Installation Error, Thrust or Shear
4	Stress Corrosion Crack	longitudinal or spiral	Corrosion & Stress, Coating Damage
5	Weld Crack	Crack in the weld area	Hydrogen Embrittlement, Fatigue
6	Rapid Crack Propagation	Linear split	Brittle Fracture, External Impact

Due to the intentional damage in the structure, Destructive Health Monitoring testing methods are not preferable as compared to non-destructive methods^9,10^. Several Non-destructive testing methods can be performed for the inspection of pipelines, including magnetic particle inspection, liquid penetrant inspection, visual testing, radiography, electromechanical impedance testing, acoustic emission testing, and ultrasonic testing. Visual testing effectively identifies surface cracks; however, its effectiveness is limited by unclear visibility, adverse weather conditions, or insufficient light. Radiography is employed to detect internal cracks within a specimen; it is a costly solution. Electromechanical Impedance Testing (EMI) uses the interaction between an electrical signal and the mechanical response of a material or structure. The response is measured as a change in impedance. Acoustic Emission Testing (AE) detects high-frequency sound waves generated by the rapid release of energy from localized sources within a material. The ultrasonic testing technique involves transmitting and receiving ultrasonic waves to detect changes caused by cracks or defects. Among the various NDT methods, Ultrasonic Testing (UT) is considered effective for the inspection of pipelines.

Ultrasonic testing uses high-frequency sound waves (ultrasound) to inspect materials for defects. The waves are introduced into the material through a transducer, and the time it takes for the sound waves to travel through the material and return is measured. Defects such as cracks, voids, or delamination will reflect sound waves differently compared to the undamaged material, which helps in identifying and characterizing the damage. It can be used for the detection of surface defects as well as for internal cracks. Primarily, two methods, pitch-catch and pulse-echo, are carried out in ultrasonic testing^11,12^. In the pitch-catch method, an actuator is used for transmitting ultrasonic waves, and sensors are used as receivers. The pitch–catch method utilizes low-frequency Lamb waves, also known as guided waves, which are effective for surface and subsurface defect detection. While in pulse-echo testing, the ultrasonic transducer operates both as a transmitter and a receiver to interpret the signal through reflection patterns. The pulse–echo method employs high-frequency waves. Piezoelectric sensors are widely used for ultrasonic testing due to their ability to convert mechanical stress into electrical signals, suitable for detecting vibrations and impacts, and offer a high sensitivity, low power requirements, provides adaptability for a wide range of materials and structures. The performance of piezoelectric sensors in SHM depends on the choice of couplant materials, which affect signal transmission and defect detection. Complementary studies demonstrated that gel-based couplants enhance signal clarity, reducing signal loss by up to $30\%$ and improving defect visibility. Real-time SHM systems utilizing piezoelectric ultrasonic transducers have been developed to monitor both internal structural damage and couplant layer quality, achieving high sensitivity to defects. These findings underscore the importance of tailoring couplant selection to specific frequency ranges and structural materials to optimize SHM performance. In composite materials, polymer-based in-situ piezoelectric sensors integrated with ML models have achieved accurate, real-time fault detection by distinguishing various fault types^13^. Furthermore, anisotropic piezoelectric sensors combining lead zirconate titanate (PZT) with electronic-grade glass fibers (EGF) have demonstrated ultra-high sensitivity to directional micro deformations, with applications in both SHM and robotic perception^14^. Recent research has investigated the shear performance of innovative composite shear connectors in steel–concrete composite structures. A compressive composite shear connector with transverse studs was studied through push-out tests and finite element analysis, demonstrating significantly higher ultimate bearing capacity than conventional connectors. A predictive model for the ultimate bearing capacity was also proposed to support structural design applications^15^. The shear behaviour of bearing–shear connectors (B-SCs) for prefabricated steel–concrete composite beams has also been examined using validated numerical models and push-out tests. The study identified key parameters influencing shear performance, including concrete strength and shear plate properties, and proposed calculation formulae for ultimate shear resistance and slip modulus^16^. In addition, thermal behaviour in advanced composite bridge structures has been investigated. A temperature gradient model for prestressed concrete beams with corrugated steel webs was developed using long-term meteorological data and a random forest algorithm, improving predictions of structural temperature gradients for engineering design^17^. Furthermore, advancements in structural monitoring techniques have been reported using computer vision based methods. A noncontact vibration measurement approach based on a line tracking algorithm was proposed for cable stayed bridges, demonstrating high accuracy in identifying cable vibration frequencies and forces for structural health monitoring^18^.

Guided wave inspection and phased array systems have significantly improved defect detection and localization in SHM. Baseline free damage imaging using ultrasonic guided waves (UGWs) with parallel arrays of PZT transducers on composite lap joints has achieved precise visualization of damage zones without requiring reference signals from healthy structures, with quadrilateral and diamond-shaped arrays proving most effective^19^. A permanently installed high-resolution ultrasonic phased array with 18 piezoceramic elements on flexible printed circuits enabled continuous monitoring of defects as small as 0.1 mm in steel, leveraging the total focusing method (TFM)^20^. Lamb wave-based SHM has advanced multi-crack detection through hybrid damage quantification algorithms, accurately identifying crack size, direction, and position in real-time^21^. A single-channel multifrequency excitation system using bandpass filter circuits has enabled cost-effective Lamb wave imaging, reducing acquisition time and data volume while maintaining precise damage localization^22^. Additionally, a unidirectional frequency-steerable acoustic transducer (FSAT) achieved $98\%$ localization accuracy in guided wave damage imaging by exploiting frequency-dependent spatial filtering^23^. Nonlinear ultrasonic imaging with dual-element transducers has improved signal-to-noise ratios for fatigue damage evaluation in thick stainless steel components^24^. Furthermore, printed ultrasound sensors made from piezoelectric composites have shown promise for non-destructive evaluation by measuring component thickness in steel and aluminum, offering a cost-effective and durable solution^25^. These advancements highlight the growing precision and scalability of guided wave and phased array.

For pipeline monitoring, FPGA-based smart SHM systems using torsional guided waves in pulse-echo mode have demonstrated excellent performance in localizing multiple defects, such as notches and mass loading, in steel pipes^26,27^. Du, Yuehao, et al.^28^ conducted a comparative study on ultrasonic C-scan imaging using piezoelectric transducers. They analyzed a Steel Pipe of 1000 mm length, 600 mm diameter, with a through-hole crack on the Surface. Their technique utilized a Bidirectional SH wave piezoelectric sensor in Pitch Catch Mode. The outcome was a Comparative study on ultrasonic C-scan imaging of composite lap joints to assess their performance. On the other hand, Shah, Jay, Said El-Hawwat, and Hao Wang^29^. focused on generating a laboratory-scale signal state for structural health monitoring. They studied a Polyethylene Pipe with a 200 mm length and a 20 mm circumferential crack on the surface. The monitoring utilized Piezo ceramic sensors in Pitch Catch Mode. In addition, Patil, S., Banerjee, S., & Tallur, S.^30^ focused on an innovative amplitude measurement approach for crack detection. They investigated a Steel Pipe of 1000 mm length, 114.6 mm diameter, with a 4 mm Notch and Mass Loss defect on the surface. Their approach used a PZT sensor in Pitch Catch Mode. The outcome demonstrated a method for signal amplitude innovation. Yang, Dan, et al.^31^ investigated a Steel Pipe of 200 mm length, 88 mm diameter, affected by corrosion. The system used a PZT sensor in Pitch Catch Mode with a CNN (Convolutional Neural Network) for signal processing. Li, Bolun, et al.^32^ offer the maximum inspection capabilities of pipelines. They examined a Steel Pipe of 1000 mm length, 610 mm diameter, with a 10 mm butt weld joint defect located on the surface. The system used a Piezoelectric micro-ultrasound sensor in pulse-echo mode. The outcome confirmed the feasibility of achieving a maximum level of inspection capability for internal defects. Yu, Sia Yee, et al.^33^ developed a non-invasive measurement method as an alternative for the inspection of pipes. They studied a Steel Pipe structure with a Longitudinal crack on the surface. The system utilized an Ultrasonic Transducer in Pulse Echo Mode. As a result, authors validate an alternative system for non-destructive testing of carbon steel pipes using non-invasive measurement methods. Al-Aribe, K. M., Mohamed, O. A., & Fakron, O. M.^34^ proposed an innovative IoT array approach for the crack detection of pipelines. They examined a Galvanized Pipe of 1000 mm length, 112 mm diameter, with a 1.9 mm circumferential surface crack. Their system utilized a Bending PZT Strip sensor in the Pitch Catch Mode, integrating IoT technology. The primary outcome was the successful implementation of an Array for Crack detection suitable for connected monitoring systems. The integration of piezoelectric and ultrasonic sensors with robotic systems has opened new avenues for automated SHM. A novel method introduced in^35^ that integrates capacitive micromachined ultrasonic transducers (CMUT) into robotic grippers to enable both proximity and tactile sensing. The on-chip integration of CMUTs facilitates tactile and reactive grasping, enhancing the gripper’s ability to interact with objects. The system demonstrates a single-channel proximity sensing capability, achieving high spatial resolution for detecting objects at distances up to 10 mm. This approach empowers robotic grippers with multimodal sensing, improving precision and adaptability in grasping tasks. An innovative robotic ultrasound diagnostic system designed by^36^ for non-destructive testing in highly variable production environments, where products vary in shape and size, often produced in small batches. The system utilizes robotic positioning to automatically adapt the ultrasound probe’s placement relative to the tested object, using laser distance measurements in a water-based diagnostic vessel. The study details the design, simulation, optimization, and verification of the system, demonstrating its capability to ensure quality control in flexible manufacturing settings through automated and precise diagnostic processes. A notable example is the integration of an ultrasonic sensor within a KUKA KR 16 robotic end effector for railway track inspection, successfully detecting internal defects in the rail web using 4–5 MHz wedged probes^37^. Awal, Md Rabiul, et al.^38^ focused on creating an automated system to detect cracks and determine the maximum defect size. They investigated aluminum pipe structures using a PVDF sensor and a DC Motor actuator in Pitch Catch Mode. The study successfully demonstrated a DC Motor crack detection method to determine the maximum achievable inspection limit. Nemati, Hamidreza, et al.^39^ explores the integration of electromagnetic acoustic transducers (EMATs) into a modular robotic gripper for non-destructive testing of tubular structures. Stainless-steel pipe structures were investigated to address circumferential and surface cracks with a length of 114.3 mm and thickness of 88.9 mm using an EMAT sensor. The system employed the Pitch Catch Mode with a Robotic manipulator to enable automation in the data acquisition pipeline’s inspection tasks.

The reviewed studies highlight significant advancements in piezoelectric sensor-based SHM, driven by innovations in sensor materials, embedded systems, signal processing, and guided wave techniques. However, challenges remain, including the need for robotic inspection technology with hybrid sensing approaches for real-time, large-scale monitoring of pipelines. The main objective of this research is to design and develop an AI-enabled SHM System integrated with a robotic gripper for the inspection of pipelines using piezoelectric smart sensors. The rest of this paper is structured as follows, brief description of the methodology and technical details of the experimental setup are provided in the Methodology section, and results and discussions are in the Results section. The Conclusion Section describes the concluding remarks and future recommendations of this work.

## Methodology

This section provides a comprehensive description of the proposed SHM system integrated with the robotic gripper for the inspection of pipelines, along with the design of the pulse echo that will be used for this work. Detailed information about the sampling materials, sensors, data acquisition, and signal processing with their installation on the experimental setup is also provided.

### Working of proposed methodology

**Fig. 2:**
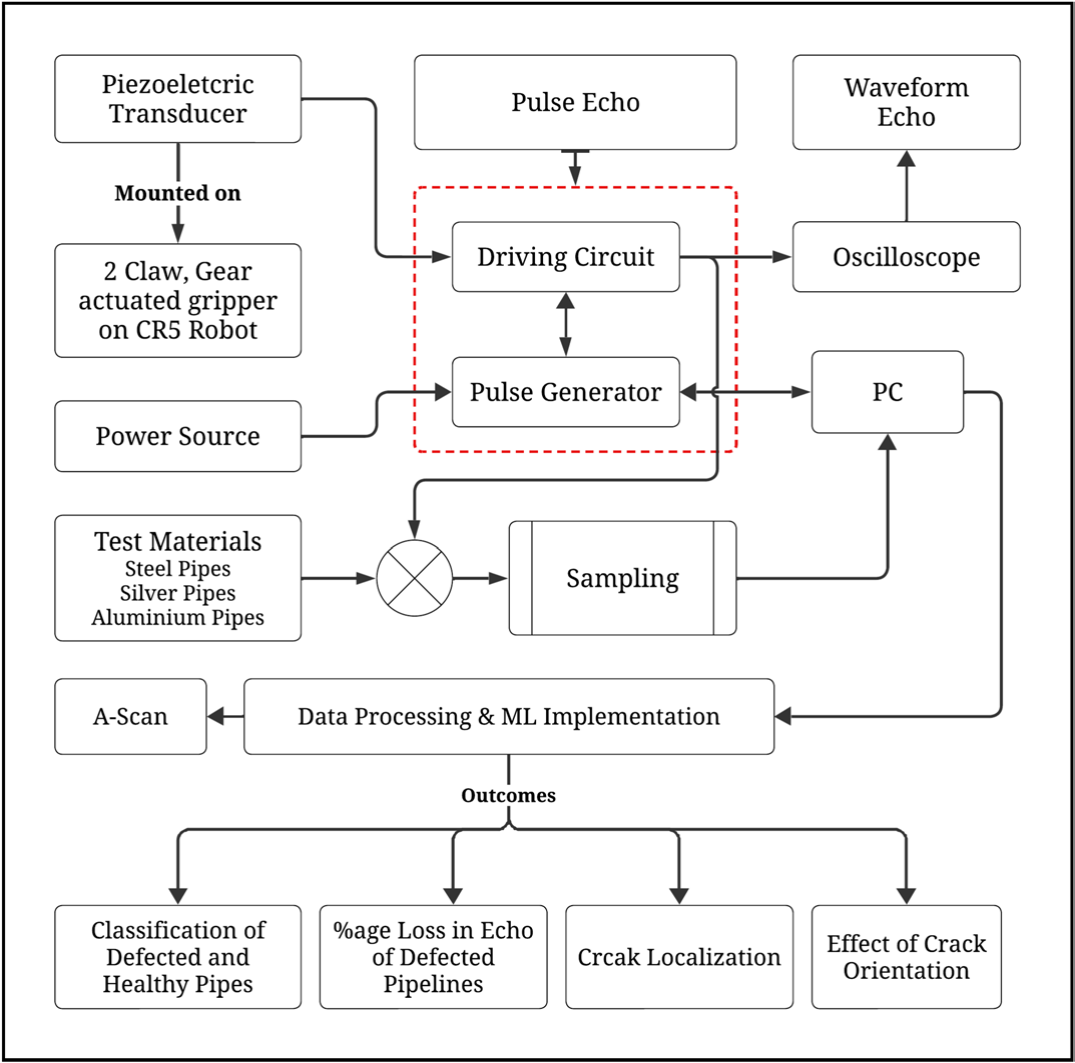
Flowchart of the proposed pipeline’s SHM system.

This section explains the working flow of the proposed system for the structural health monitoring (SHM) of pipelines, as shown in the flowchart Fig. 2. The process starts with piezoelectric transducers (sensors) attached to a robotic gripper. This gripper is mounted on a CR5 robotic arm. To ensure consistent signal transmission and to prevent noise from inconsistent coupling or clamping forces, the Piezo sensors are mounted on the claws of the gripper using flexible, compressible 2mm adhesive tape. This setup functioned as a shock absorber and vibration damper, maintaining a consistent contact pressure and preventing the false positives or signal spikes that often result from excessive clamping force. A coupling material is used to avoid signal losses between the sensor and the test material surface. The pulse-echo system includes a pulser (to generate signals), a driving circuit, and a power supply. The returning echo signals are displayed on an oscilloscope and sent to a computer (PC) for data collection and storage. The recorded signals are then processed and visualized using MATLAB and Google Colab. These tools help analyze the data to detect defects in the pipeline, measure signal loss percentage, locate cracks, and evaluate the effect of the direction (orientation) of cracks in the reflected signals by the integration of AI and ML algorithms. The complete hardware configuration and interfacing of the experimental setup are illustrated in Fig. 7.

### Pulse Echo

**Fig. 3:**
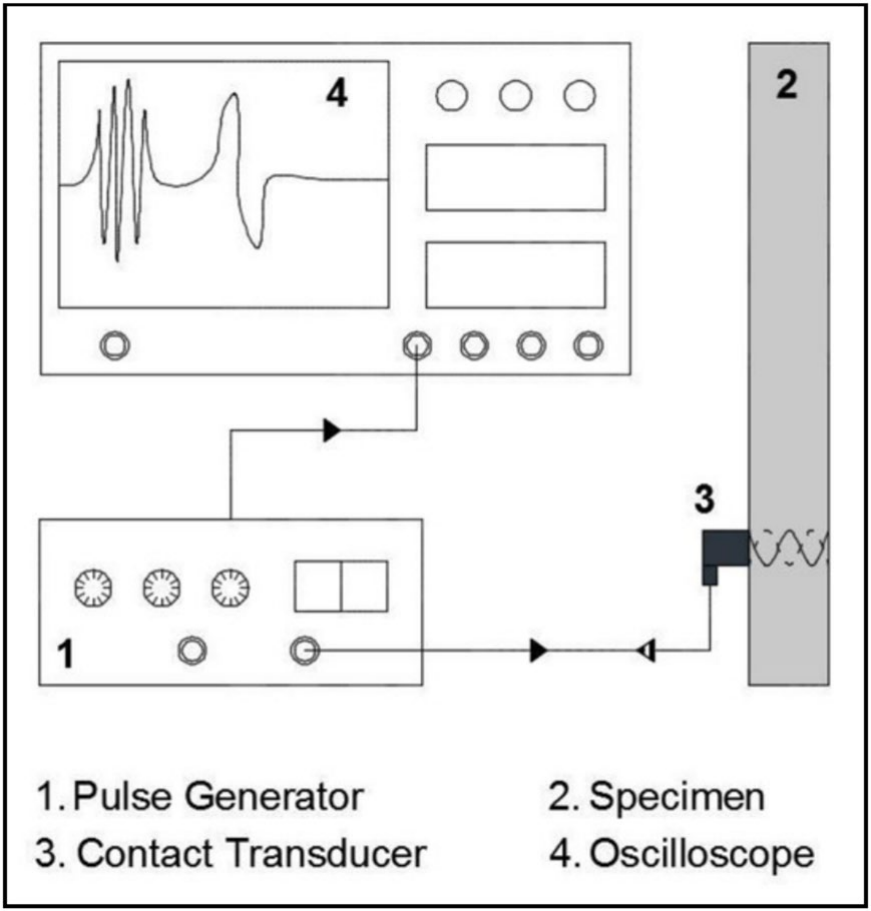
Components involved in pulse-echo UT technique^40^.

The Pulse Echo UT testing method requires only a single ultrasonic transducer, which can operate both as a transmitter and receiver to interpret the signal through reflection patterns. These are the following major components: pulse generator, ultrasonic transducer, test specimen material, and computer-based system or oscilloscope for displaying echo signals, involved in the Pulse Echo UT technique as described in Fig. 3. The pulse generator produces an electrical pulse, which the ultrasonic transducer converts into an acoustic wave that propagates into the test material. Upon encountering a defect or boundary, the wave reflects as an echo, which is detected by the transducer. This echo is then converted back into an electrical signal and processed by the computer or oscilloscope to identify, locate, and characterize damage within the structure.

### Piezoelectric ultrasonic transducer

The piezoelectric circular disc sensor is used as an ultrasonic transducer in this work that exhibits a dual functionality, serving both as a sensor and an actuator due to the direct and inverse piezoelectric effect. In actuation mode, it converts electrical signals into mechanical vibrations to generate ultrasonic waves (inverse piezo effect). In sensing mode, it converts incoming mechanical vibrations from reflected echoes back into electrical signals for further analysis (direct piezo effect). The key specifications of the piezoelectric ultrasonic transducer are summarized in the Table 2. The excitation frequency of 33 kHz for the piezoelectric ultrasonic transducer is selected based on the impedance analysis through an LCR meter.

**Table 2:** Specifications of piezoelectric transducer.

Parameter	Specification
Geometry	Circular Disc
Outer Diameter	27 mm
Inner Diameter	18 mm
Thickness	0.3 mm
Plate Material	Brass
Operating Temperature	$-20^\circ$C to $+70^\circ$C
Resonance Frequency	2.9-3.5 kHz
Capacitance	2.5 nF (at 1 kHz)
Ceramic Material	Lead Zirconate Titanate

### Pulse generator

**Fig. 4:**
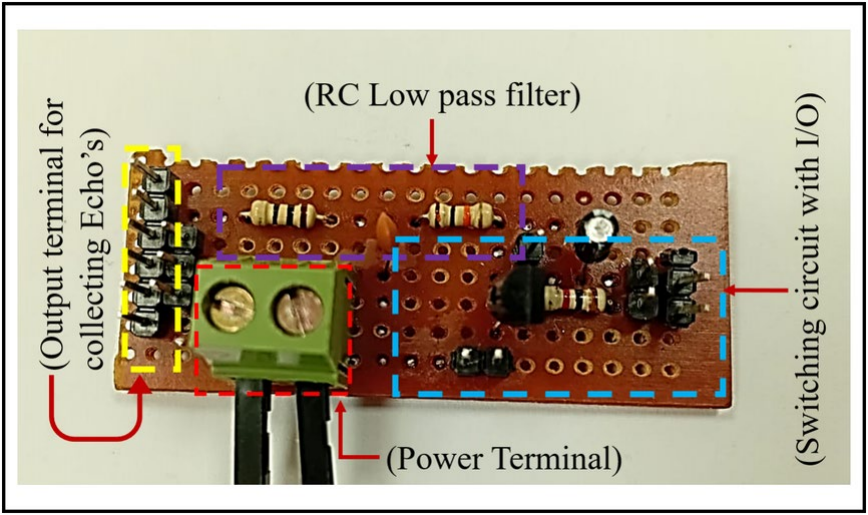
Schematic of the driving circuit.

The pulse generator, the heart of the experimental setup, is responsible for generating electrical pulses at a fixed frequency that further excite the ultrasonic transducer for the generation of ultrasonic waves. For this setup, an Arduino Uno microcontroller, combined with a driving circuit, is employed. The Arduino Uno generates pulses at the selected excitation frequency. The driving circuit incorporates a BJT transistor functioning as a switch to toggle the piezoelectric transducer between actuation and sensing modes. A compact schematic of the driving circuit is provided in the accompanying Fig. 4. This configuration maintains the transducer in actuation mode for 5 microseconds to generate the pulse, followed by 33 microseconds in sensing mode to capture and record the reflected echoes from the crack.

The driving circuit is powered via the Power source terminals. The Switching I/O terminals (right side) connect to an Arduino Uno microcontroller, which serves as the pulse generator. The core functionality is handled by the Switching circuit (blue box), which incorporates a BJT transistor to function as a switch. The received echoes are directed to the Output terminals for collecting Echo’s (yellow box), and an RC low-pass filter (purple box) is included to condition the signal and to avoid high-frequency noise.

### Test specimens

**Fig. 5:**
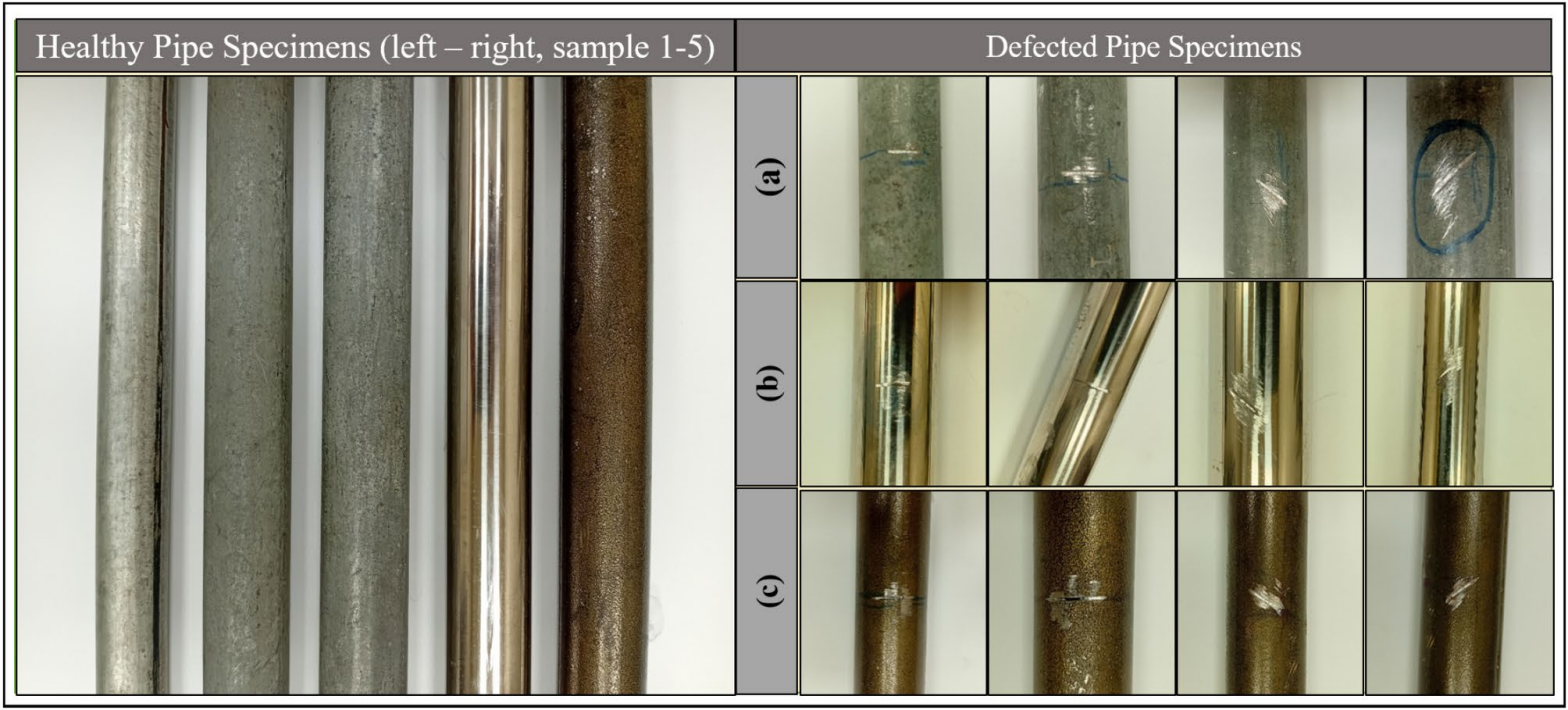
Test specimens employed during the experimentation for the SHM of pipelines.

Circular pipes of Steel, Silver, and Aluminium materials shown in Fig. 5 are used as test specimens in this work to evaluate the system’s performance across materials. The steel pipe samples included four different geometries with respect to their nominal diameter and thickness, given in Table 3, Samples 1-4, as compared to the Silver pipe sample 5 and Aluminium pipe Sample 6. These multiple samples allow for a comparative analysis of defect detection across metallic pipe structures commonly encountered in industrial applications. The physical dimensions of the samples varied, with Outer Diameters ranging from 20.5 mm to 27 mm and Thicknesses from 0.55 mm to 2.25 mm.

### Sensor geometry & pipe contact area

**Fig. 6:**
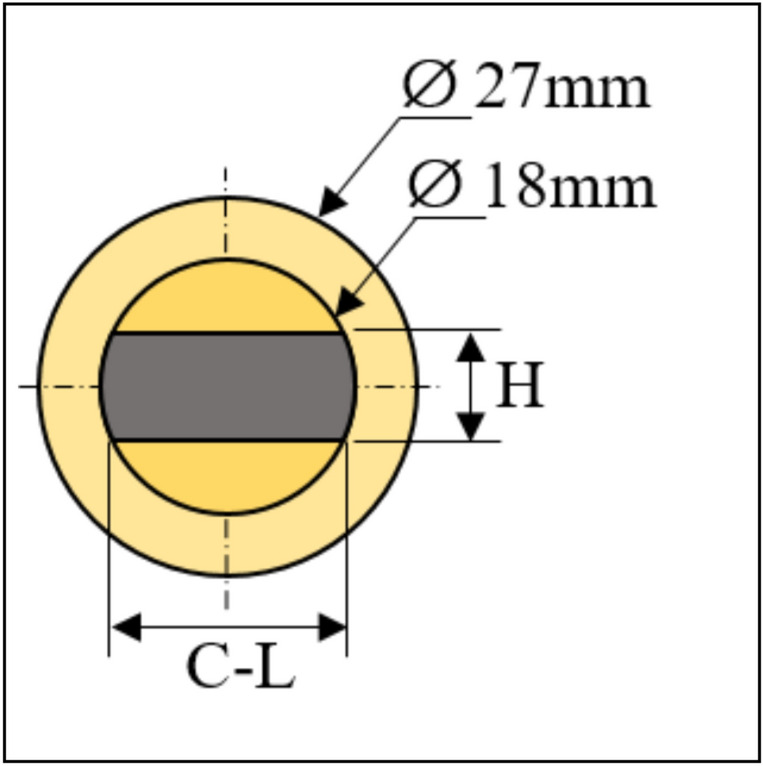
Sensor geometry and effective contact area on the curved pipe surface.

In this study, we have used a circular piezoelectric disc sensor with a 27 mm outer diameter and an active crystal diameter of 18 mm. The detailed dimensions and specifications of the sensor are provided in Table 2 of the manuscript. Although the sensor has a flat surface and the pipe is curved, the contact area is not a theoretical line. Because the sensor diameter is relatively small compared to the pipe diameter, the effective contact region becomes a finite rectangular area with curved (sector-shaped) edges, rather than a line. This contact geometry is illustrated in Fig. 6.

The contact region is defined by two symmetric chord lengths (C-L) at a height (H) from the sensor center, with curved sector edges resulting from the pipe curvature. Specifically, for a 20.5 mm diameter pipe, C-L = 17.5 mm and H = 7.5 mm; for a 25.4 mm diameter pipe, C-L = 17.0 mm and H = 8.0 mm; and for a 27.0 mm diameter pipe, C-L = 16.5 mm and H = 9.0 mm. These values confirm that a stable and repeatable contact area exists between the sensor and the pipe surface.

### Data acquisition & processing

A PC and oscilloscope are utilized for data visualization and initial signal monitoring. The oscilloscope provides a real-time display of echo signals, while the PC enables storage, processing, and detailed analysis of the acquired data. During the sensing mode of the piezoelectric transducer, the raw echo signal is passed through an RC low-pass filter with a -3 dB cutoff frequency of 34.6 kHz to attenuate high-frequency noise while preserving the relevant ultrasonic content. The filtered data is then stored in CSV format.

The CSV data is imported into MATLAB for visualization and processing. A moving average filter with a window size of 5 is applied to smooth the signal and reduce noise.

**Fig. 7:**
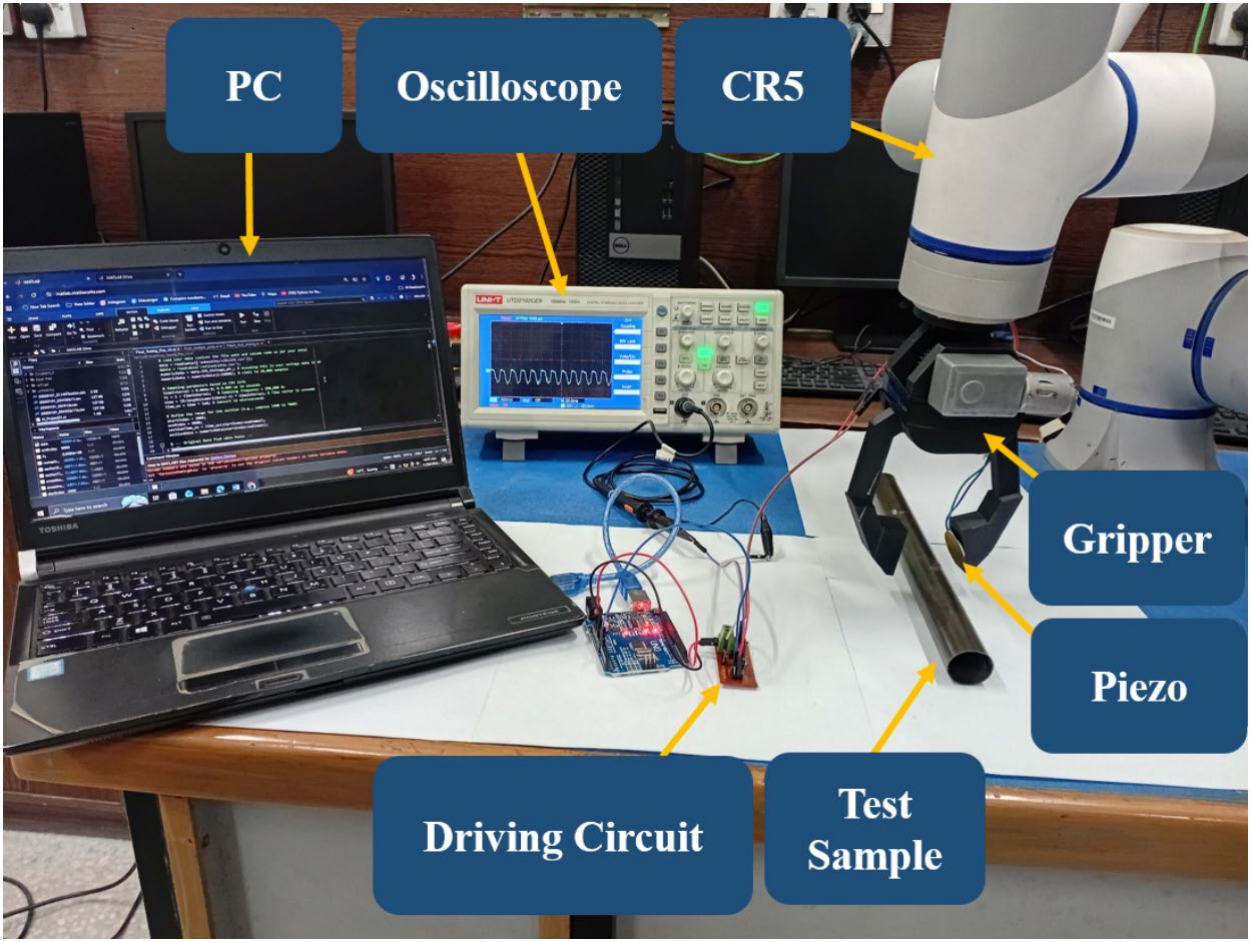
Experimental setup for pipeline inspection.

A-scans (Amplitude Scan) of the acquired signals are plotted using MATLAB to represent the amplitude of the echo versus time. An A-scan is a one-dimensional display showing signal intensity over time from a single transducer position, ideal for detecting defects along a straight path. In contrast, a B-scan (Brightness Scan) compiles multiple A-scans into a two-dimensional cross-sectional image, plotting amplitude as brightness or color along a scan line, which is useful for visualizing defect shape and location in a plane but requires transducer movement or array setups. To enhance the practicality and automation of the Structural Health Monitoring (SHM) system, the piezoelectric transducer is integrated with a two-claw, geared mechanism robotic gripper mounted on a CR5, 6 DOF robotic manipulator as shown in Fig. 7. The piezo sensor is attached to the claws, enabling precise positioning and contact with the test structures during inspection. The integration of robotic Arms for Measurement offers significant advantages in terms of testing throughput and spatial precision, though it introduces specific implementation challenges. The primary advantage is the elimination of human-induced variability; the robot ensures that the Piezo sensor is positioned with sub-millimeter accuracy and consistent contact pressure across all iterations. However, practical challenges included managing the mechanical vibrations and electromagnetic interference (EMI) generated by the robot’s actuators. These were successfully mitigated by operating the system at 50% speed ($1.5\text { m/s}$) and utilizing a specialized, vibration-dampening adhesive interface for sensor mounting. This robotic approach ultimately provides a more reliable framework for high-volume ultrasonic testing compared to manual methods, as it maintains a constant clamping-to-signal ratio that is critical for accurate echo acquisition. This setup not only automates the pulse-echo process but also improves repeatability, reduces human error, and extends applicability to hard-to-reach structural areas in real-world scenarios.

To minimize the influence of random errors and ensure high reliability, several rigorous measures were implemented during the experimentation. First, environmental variables were stabilized by conducting all tests within a controlled temperature range of $25^\circ$C and constant humidity. To ensure statistical consistency, each sample was tested four times under identical conditions, and representative data were analyzed to validate the experimental outcomes. Mechanical noise and vibrations were mitigated by operating the robotic manipulator at $1.5\text { m/s}$–exactly 50% of its maximum speed, ensuring a stable platform for signal acquisition. Furthermore, to prevent noise from inconsistent coupling or clamping forces, the Piezo sensors were mounted on the claws of the gripper using flexible, compressible 2mm adhesive tape. This setup functioned as a shock absorber and vibration damper, maintaining a consistent contact pressure and preventing the false positives or signal spikes that often result from excessive clamping force. A couplant material is used to minimize signal losses between the sensor and the test material surface.

**Table 3:** Specifications of test specimens employed in the experimental study.

Sample	Material	OD (mm)	T (mm)	L (mm)	$\rho \; (kg/m^3)$	C (m/s)	Z $(kg/m^2\cdot s)$
1	Steel	27	2.25	280	7,850	5,960	$46.8 \times 10^6$
2	Steel	25.4	1.7	300	7,850	5,960	$46.8 \times 10^6$
3	Steel	20.5	1.8	270	7,850	5,960	$46.8 \times 10^6$
4	Steel	25.4	1.7	300	7,850	5,960	$46.8 \times 10^6$
5	Silver	25.4	0.55	300	10,490	3,650	38.3 $\times 10^6$
6	Aluminium	25.4	0.9	300	2,700	6,420	$17.3 \times 10^6$

OD:outer diameter; T:thickness; L:length of pipe; $\rho$:density of material; C:speed of sound; and Z:acoustic impedance.

## Results and discussion

**Fig. 8:**
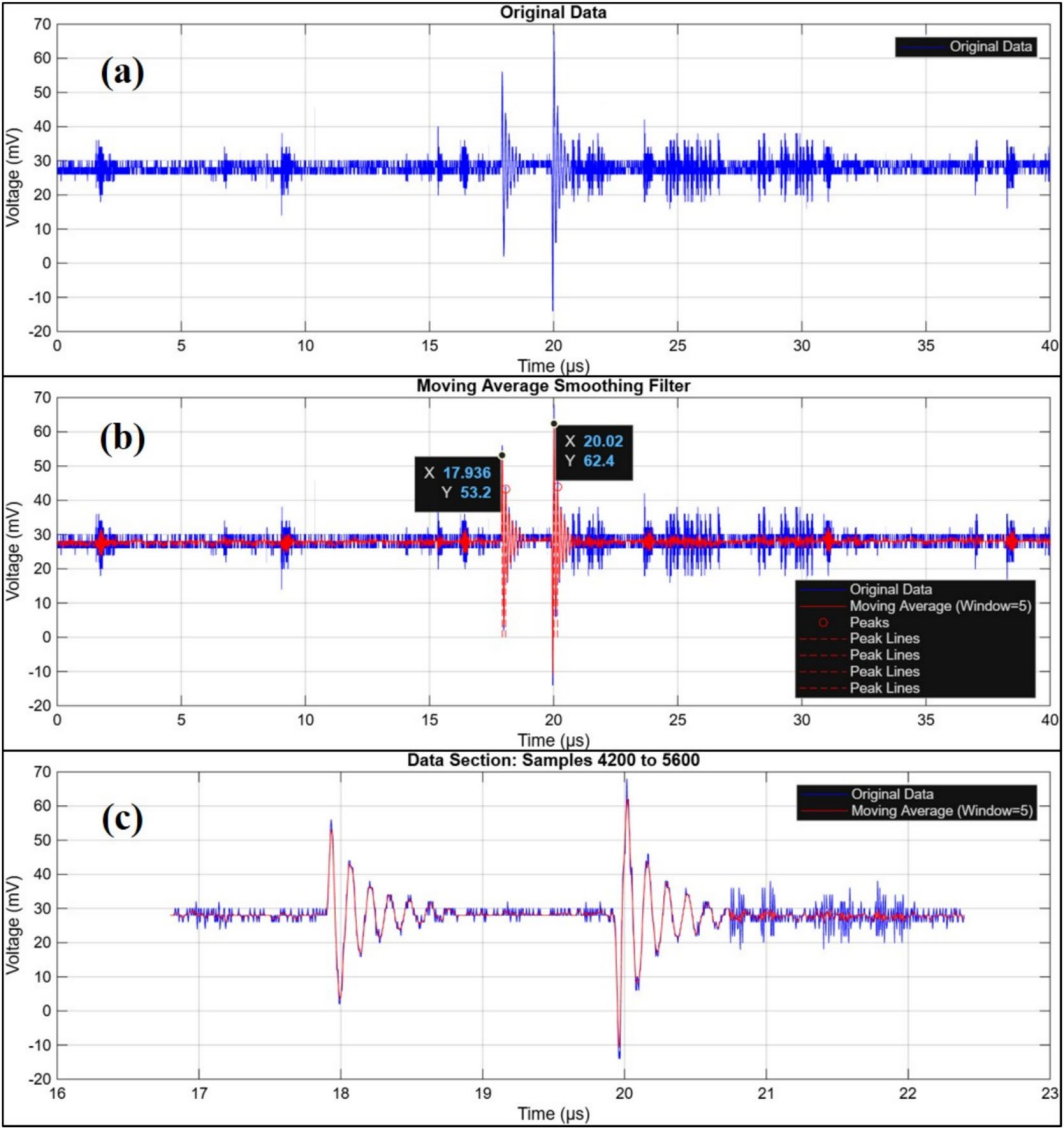
(**a**) Raw acquired signal, (**b**) normalized data, and (**c**) sectional plot of normalized data presenting reflected echo.

To evaluate the performance of the proposed SHM pulse echo system, experiments are performed on three categories of circular pipes with materials (steel, silver, and aluminum), each with varying outer diameters and wall thicknesses. The experimental setup consists of a piezoelectric ultrasonic transducer, mounted on the claws of a robotic gripper that facilitates the SHM process by collecting data, which was subsequently stored in CSV format. The collected data underwent pre-processing using low-pass and moving average filters implemented in MATLAB software and plotted in the form of A-Scans containing multiple peaks of different amplitudes, defining different modes of ultrasonic waves. The piezoelectric prob, positioned at 35$\%$ of the pipe length, generates longitudinal guided waves in pulse-echo mode and captures reflected echoes from the pipe edges and cracks. The echoes are denoted as: TP_1_ (time of first peak), also known as L(0,1) mode with amplitude V_1_, and TP_2_ (time of second peak), called as L(0,2) mode with amplitude V_2_. The L(0,1) mode is faster, arrives earlier, but with lower amplitude due to higher attenuation, while the L(0,2) mode is slower and shows larger amplitude due to better energy coupling at the tested frequency-thickness product. Figure 8 illustrates the processed A-scan results containing time on x-axis and voltage amplitude of reflected echo on y-axis, where sub-figure (a) presents the original acquired data, sub-figure (b) shows the normalized data after applying the moving average filter, and sub-figure (c) highlights a sectional plot presenting the reflected echo from the edges of the test specimen.

Additionally, various test cases with and without couplant are examined in this work based on the combinations of specimen properties, including material type, diameter, thickness, and the orientation of induced surface cracks. Case 1 describes the Testing of healthy Steel Pipe specimens (same material) with a difference in diameter and wall thickness of the pipe. Case 2 explains the Testing of healthy Steel, silver, and Aluminium Pipe specimens (different materials) with the same diameter and length of pipe but with a difference in the wall thickness. While case 3 describes the Testing of defected Steel, silver and Aluminium Pipe specimens (different material) with induced crack length (1cm and 1.5cm), perpendicular to the longitudinal axis of pipe and case 4 Testing of defected Steel, silver and Aluminium Pipe specimens (different material) with induced crack length (1cm), at orientation of $45^\circ$, $90^\circ$ and $135^\circ$ to the longitudinal axis of pipe.

The experimental parameters are designed based on the comparative analysis of previous studies. All the studies listed in Table 7 focus on SHM-based NDT inspection of circular pipes, with each study using a different design approach. To provide a comprehensive investigation and to extend the existing research, this study was designed by considering the limitations of previous work. The experimental parameters were selected to achieve a clear understanding of ultrasonic testing behavior in pipes made from different materials, showing that the acoustic impedance of the material is a dominant factor. Different pipe lengths, diameters, and thicknesses were used because most previous studies were limited to a single material and fixed pipe dimensions. While Study^39^ experimentally validated pipes of the same length with three different diameters and identical thickness. To introduce greater diversity and improve understanding, this study includes pipes with varying lengths, diameters, and thicknesses. The main focus of this work is pulse-echo-based SHM testing using a piezoelectric ultrasonic transducer to detect circumferential cracks. The experimental results demonstrate the effectiveness of the pulse-echo SHM system in detecting structural anomalies across different pipe configurations by validating the proposed methodology. By analyzing the processed data, clear distinctions in the reflected echoes were observed, corresponding to variations in material properties and induced defects. These findings validate the robustness of the developed setup for monitoring circular pipes under diverse conditions.

### Case 1

In this case, the experimental results for ultrasonic wave propagation in healthy steel pipe specimens, focusing on the effects of varying outer diameter and the wall thickness of the pipe, with sanitizer as a couplant. During the testing on the healthy steel pipes without couplant the amplitude of reflected echo, for sample 1 $V_1$=53.2 mV at time 17.936 $\mu s$, and $V_2$=62.4 mV at time 20.02 $\mu s$, for sample 2 $V_1$=67.84 mV at time 15.908 $\mu s$, and $V_2$=71.68 mV at time 20.08 $\mu s$, and for sample 3 $V_1$=66.8 mV at time 11.624 $\mu s$, and $V_2$=76.4 mV at time 20.232 $\mu s$ are recorded. By reducing the pipe diameter time of reflected echoes TP_1_ and TP_2_ decreases as a result of faster wave travel due to increased confinement, and the amplitude V_1_ and V_2_ increase due to reduced dispersion and attenuation.

The amplitude of reflected echo, for sample 1, the $V_1$ increased from 53.2 mV to 54.4 mV, and the $V_2$ value increased from 62.4 mV to 65.6 mV, for sample 2, the $V_1$ value increased from 67.84 mV to 69.44 mV, and the $V_2$ value increased from 71.68 mV to 74.88 mV, for Sample 3, the $V_1$ value increased from 66.8 mV to 72 mV, and the $V_2$ value increased from 76.4 mV to 82.82 mV. As a result, the couplant improves signal quality by minimizing impedance mismatch, resulting in higher V_1_/V_2_ and slightly adjusted TP_1_/TP_2_.

The amplitude of reflected ultrasonic echoes is directly proportional to the couplant efficiency (C), as the sanitizer couplant enhances signal transmission by reducing impedance mismatch, and to mode excitability ($E_m$), with L(0,2) showing higher amplitude due to better energy coupling at the tested frequency-thickness product. Conversely, V_1_ and V_2_ are inversely proportional to the diameter of the pipe (D), as smaller diameters increase amplitudes by enhancing wave confinement and reducing attenuation ($\alpha$), which scales with diameter. The amplitudes are also inversely proportional to wall thickness (t), as slight increases reduce V1 and V2 due to changes in the frequency-thickness product affecting mode dispersion. Mathematically, Eq. 1 expresses the reflected echoes of a pulse-echo SHM system in detecting structural anomalies across different pipe configurations of the same material.

1 $$\begin{aligned} Echo\; (amplitude) \propto \frac{C \cdot E_m}{D \cdot t \cdot \alpha (D, t)} \end{aligned}$$

**Fig. 9:**
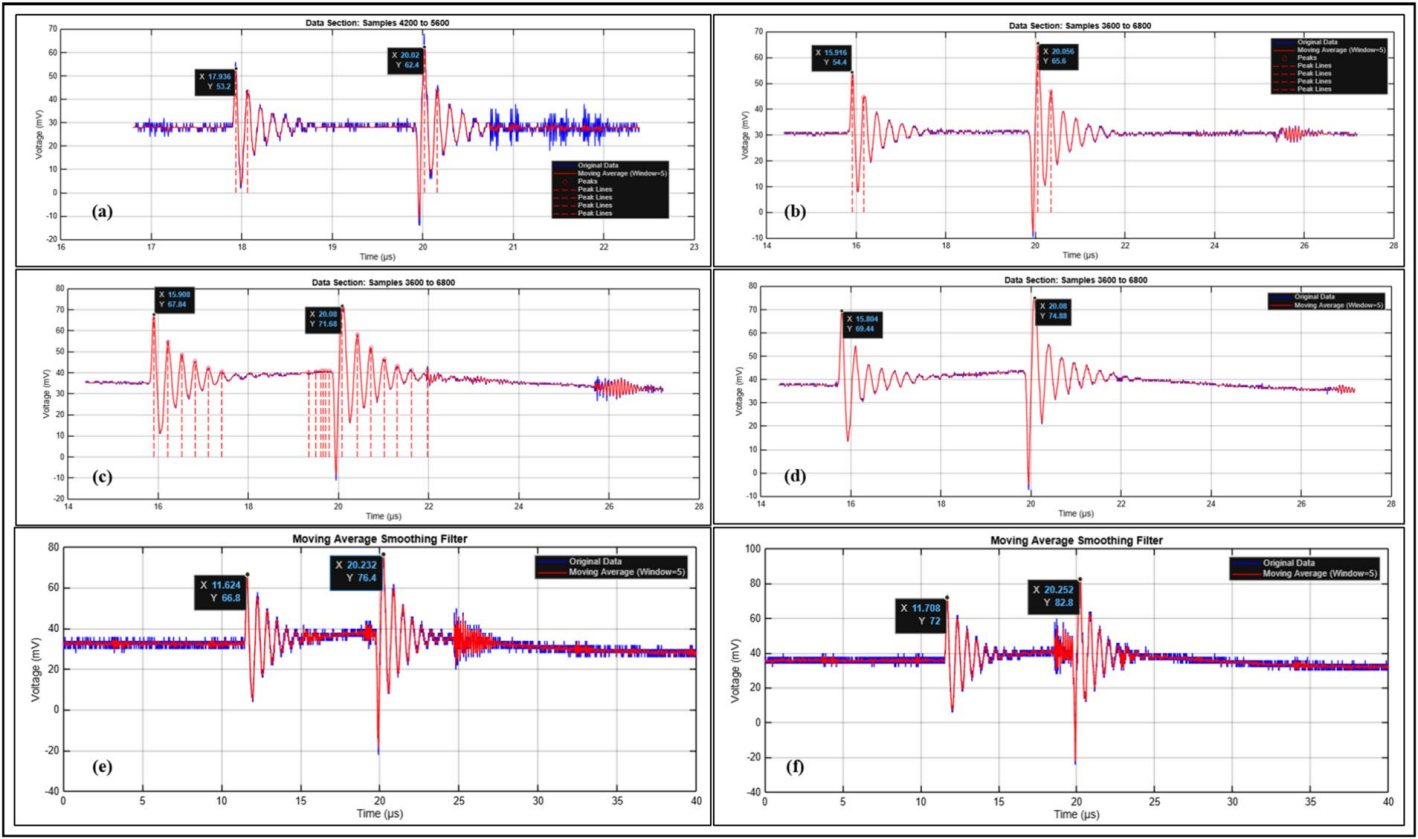
Illustration of the processed data of Samples 1, 2, and 3. Sample 1: (**a**) Sectioned data (without couplant); (**b**) Sectioned data (with couplant). Sample 2: (**c**) Sectioned data (without couplant); (**d**) Sectioned data (with couplant). Sample 3: (**e**) Normalized data (without couplant); (**f**) Normalized data (with couplant). Here, the couplant visibly suppresses noise, leading to more distinct echo signals.

Figure 9 illustrates the A-scans of healthy samples. Sub-figure (a-b) shows the A-scan of sample 1, (c & d) for sample 2, and (e & f) for sample 3.

### Case 2

In this case, the experimental results for the healthy pipe specimens made from different materials, steel, silver, and aluminum, with the same outer diameter but varying thicknesses, are tested. During the testing on the healthy steel, silver, and aluminum pipes without couplant the amplitude of reflected echo, for sample 2 $V_1 = 67.84$ mV, at time $15.908 \mu s$ and $V_2 = 71.68$ mV at time $20.08 \mu s$, for sample 4 $V_1 = 73.12$ mV, at time $11.832 \mu s$ and $V_2 = 76.16$ mV at time $20.2 \mu s$, for sample 5 $V_1 = 54.8$ mV, at time $11.504 \mu s$ and $V_2 = 67.6$ mV at time $20.192 \mu s$ are recorded. On the other hand by utilizing couplant the amplitude of reflected echo, for sample 2 $V_1 = 69.44$ mV, at time $15.804 \mu s$ and $V_2 = 74.88$ mV at time $20.08 \mu s$, for sample 4 $V_1 = 74.4$ mV, at time $11.776 \mu s$ and $V_2 = 80.96$ mV at time $20.2 \mu s$, for sample 5 $V_1 = 62.4$ mV, at time $11.576 \mu s$ and $V_2 = 73.2$ mV at time $20.236 \mu s$ are recorded. Silver consistently produced the highest V_1_ and V_2_, followed by steel and aluminum, due to material-specific acoustic properties, including acoustic impedance and wall thicknesses of pipe. Acoustic impedance (Z) is given by Z = $\rho \cdot$ c, where $\rho$ is the density and c is the speed of sound. Acoustic properties of the materials tested (steel, silver, and aluminum) in this work are given in Table 3.

**Fig. 10:**
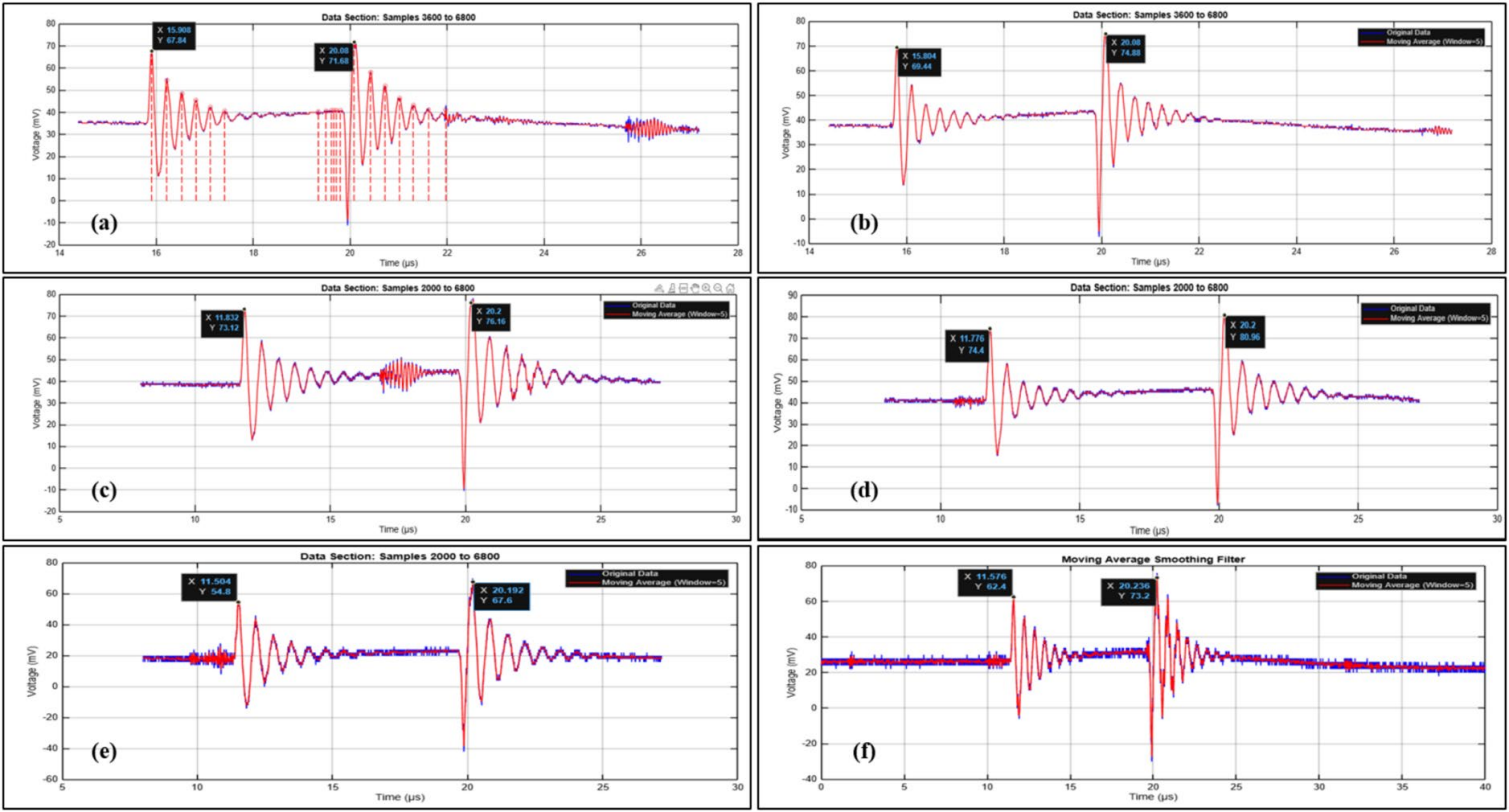
Illustration of the processed Sectioned data with and without couplant for Sample 2 in (**a** & **b**), Sample 4 in (**c** & **d**), and for Sample 5 in (**e** & **f**).

The voltage amplitude depends on energy transmission efficiency, governed by the excitability and attenuation of L(0,1) and L(0,2) modes. Silver’s high density and thin profile (0.55 mm) minimize attenuation and enhance mode excitability at the frequency-thickness product (fd), leading to stronger L(0,1) and L(0,2) reflections, with V_2_ typically higher due to better coupling of the L(0,2) mode. Steel, with higher impedance but thicker walls (1.7 mm), experiences more attenuation, reducing L(0,1) and L(0,2) amplitudes. Aluminum’s low impedance and moderate thickness (0.9 mm) result in the weakest signals due to less efficient energy reflection. The L(0,1) mode’s faster group velocity yields earlier TP_1_, while L(0,2)’s slower velocity causes later TP_2_, with times inversely proportional to material sound speed and slightly reduced by couplant due to improved transmission. Figure 10 illustrates the A-scans of healthy steel, silver, and aluminium pipe samples tested with and without couplant material. Sub-figure (a & b) shows the A-scan of steel sample pipe, (c & d) for silver sample pipe, and (e & f) for aluminium sample pipe.

### Case 3

**Fig. 11:**
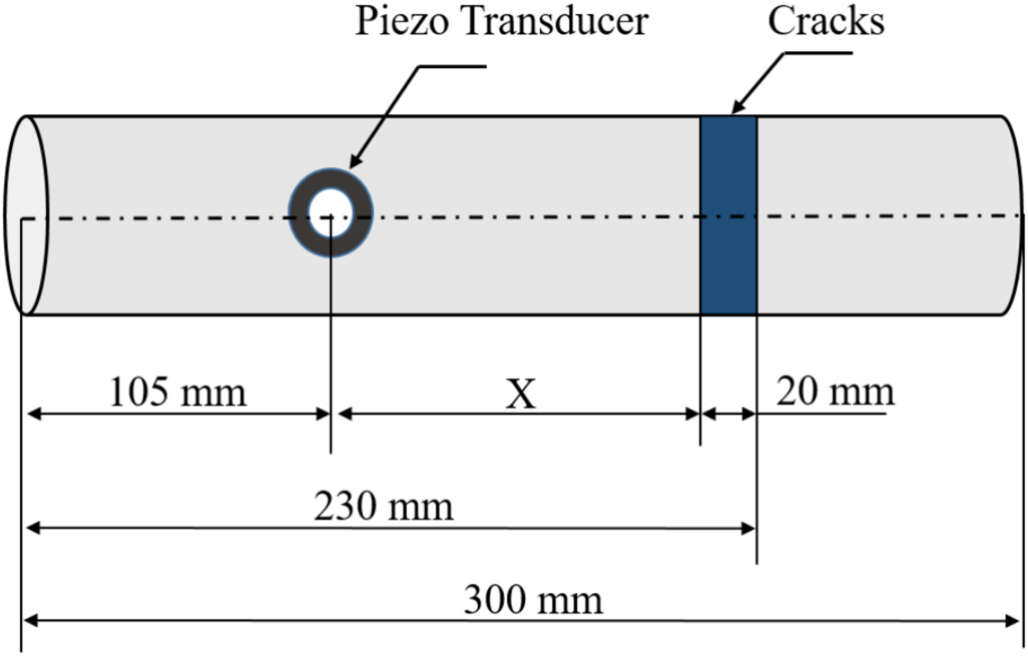
Schematic diagram of pipeline test specimen with labeled dimensions, piezoelectric transducer, and cracks placement.

This case describes the experimental results from ultrasonic testing of defective pipe specimens made of different materials (steel, silver, aluminum)11. All specimens have the same outer diameter but vary in wall thickness; key specifications are described in the Table 3. They include surface circumferential cracks of 1 cm and 1.5 cm in length (0.5 mm depth, 90-degree orientation) shown in Fig. 11, to examine their influence on ultrasonic wave propagation.

Silver pipes shows the highest V_1_ and V_2_ values due to the high density ($\rho \approx 10{,}490 \, \text {kg/m}^3$), moderate sound speed ($c \approx 3{,}650 \, \text {m/s}$), and thin wall (0.55 mm), which cut down attenuation and boost excitation of the L(0,1) and L(0,2) modes at the chosen frequency-thickness product (fd). Steel pipes have high acoustic impedance ($Z \approx 46.8 \times 10^6 \, \text {kg/(m}^2 \cdot \text {s)}$) but a thicker wall (1.7 mm), so attenuation rises. Aluminum pipes have low impedance ($Z \approx 17.3 \times 10^6 \, \text {kg/(m}^2 \cdot \text {s)}$) and a medium thickness (0.9 mm), giving the weakest echoes. For the 1 cm crack length with couplant A-scan of silver pipes shows V_1_ = 68.80 mV, V_2_ = 72.80 mV; for steel pipes V_1_ = 59.60 mV, V_2_ = 64.80 mV; and for aluminum pipes V_1_= 54.00 mV, V_2_ = 58.40 mV. While in the case of a crack length of 1.5 cm, the amplitude signals drop more than in the 1 cm crack. Scattering and mode conversion at the crack break up the L(0,1) and L(0,2) waves.

**Fig. 12:**
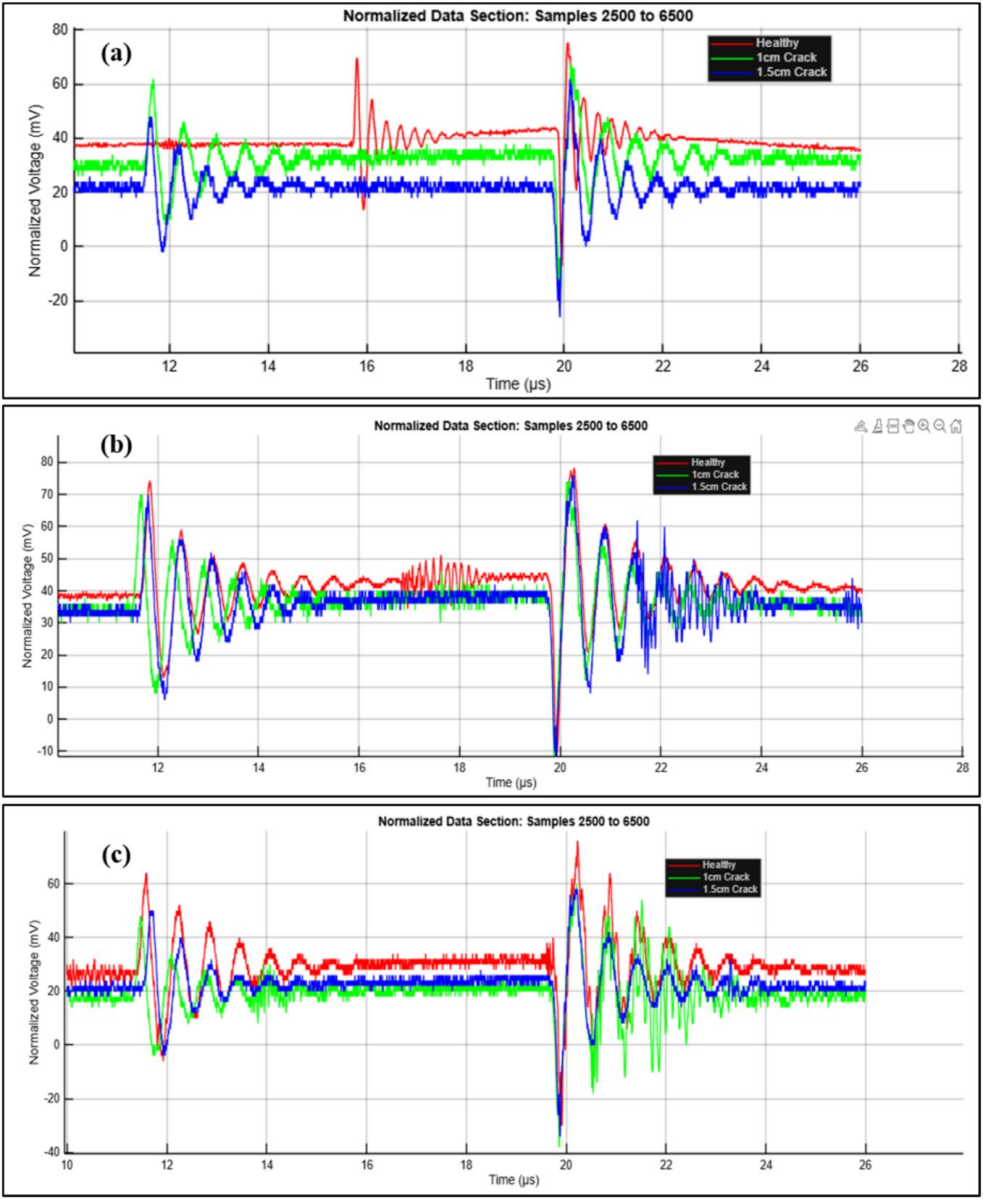
Normalized ultrasonic waveforms for (**a**) steel, (**b**) silver, and (**c**) aluminum pipes, comparing healthy (red), 1 cm crack (green), and 1.5 cm crack (blue) conditions.

Figure 12 shows normalized ultrasonic signals for healthy and defective pipe samples across the three materials. Subfigure (a) compares steel pipes, with a signal loss of 9.5% for the 1 cm crack and 17.5% for the 1.5 cm crack relative to the healthy sample. Subfigure (b) presents silver pipes, where the 1 cm crack drops the peak by 3.1% and the 1.5 cm crack by 5.6% compared to the intact pipe. Subfigure (c) covers aluminum pipes, showing a 21% reduction with the 1 cm crack and 23% with the 1.5 cm crack against the healthy baseline. In each plot, the healthy trace (red) sits highest, the 1 cm crack (green) sits lower, and the 1.5 cm crack (blue) shows the weakest response, with clear drops in amplitude. These %age signal losses were calculated from peak amplitudes relative to each healthy case as shown in Table 4.

**Table 4:** Percentage signal loss for 1 cm and 1.5 cm cracks at various orientations compared with healthy pipe samples.

Sr No.	Sample	1cm crack at $45^\circ$	1cm crack at $90^\circ$	1cm crack at $135^\circ$	1.5cm crack at $90^\circ$
1	Steel pipe	1.60 %	9.5%	1.60 %	17.5%
2	Silver pipe	1.79%	3.1%	1.79%	5.6%
3	Aluminium pipe	18.42%	21%	19.74%	23%

### Case 4

This case briefly describes the experimental results of defective pipe specimens made from steel, silver, and aluminum. All pipes have the same outer diameter of 25.4 mm but different wall thicknesses (steel: 1.7 mm, silver: 0.55 mm, aluminum: 0.9 mm). Each pipe has a 1 cm surface crack (0.5 mm depth) placed at three different orientations ($45^\circ$, $90^\circ$, and $135^\circ$).

The main crack angle on the propagatgoal is to see the effect of ion of the ultrasonic wave. Tests with and without sanitizer couplant show that couplant slightly reduces echo arrival times (TP_1_for steel at $135^\circ$ crack: 11.724 ${\upmu }$s without vs. 11.624 ${\upmu }$s with) and boosts amplitudes by filling air gaps and improving acoustic coupling. Silver shows higher amplitude peaks compared to steel and aluminum. Crack orientation also affects the signal strength, as examined cracks at $45^\circ$ and $135^\circ$ give stronger echoes than $90^\circ$ cracks because slanted faces cause less back scattering and mode conversion, letting more coherent energy travel through the longitudinal modes. Steel, despite its high acoustic impedance, shows more attenuation from its thicker wall, while aluminum’s low impedance leads to the weakest signals.

**Fig. 13:**
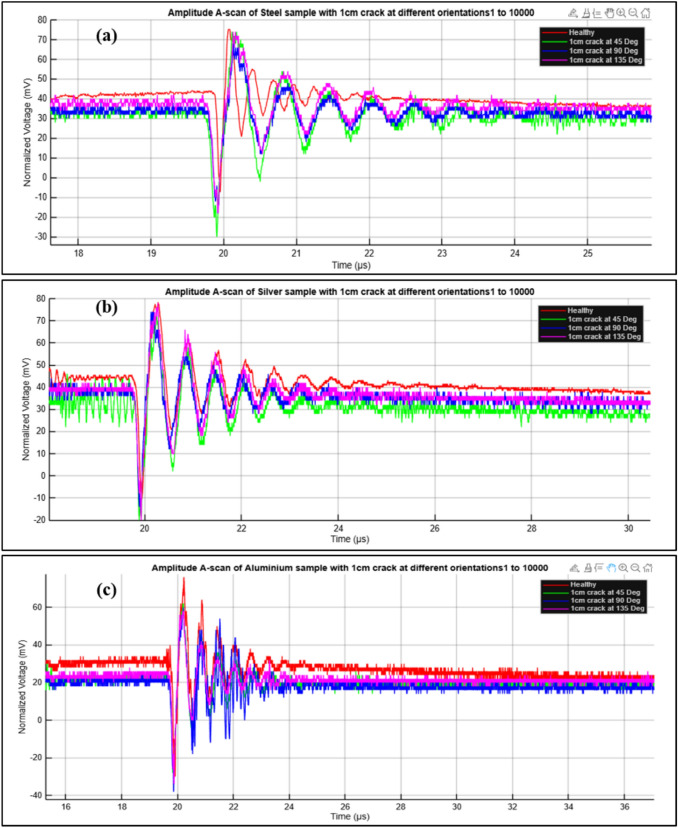
Normalized ultrasonic A-scans for (**a**) steel, (**b**) silver, and (**c**) aluminum pipes with 1 cm crack at $90^\circ$ (blue), $45^\circ$ (green), $135^\circ$ (purple), compared to healthy (red).

 The near total reflection ($R \approx 1$) at the material-air interface holds for all metals, but crack angle drives scattering losses, with $90^\circ$ orientation causing maximum disruption and oblique angles keeping signal integrity. Figure 13 shows normalized A-scan waveforms for 1 cm cracked pipes at different crack angles ($45^\circ$, $90^\circ$, and $135^\circ$) compared to healthy pipes for all three materials. Subfigure (a) compares steel pipes, with signal loss of 9.5% at $90^\circ$ crack, 1.60% at $45^\circ$ crack, and 1.60% at $135^\circ$ crack relative to the healthy sample. Subfigure (b) presents silver pipes, where the $90^\circ$ crack drops the peak by 3.1%, the $45^\circ$ crack by 1.79%, and the $135^\circ$ crack by 1.79% compared to the intact pipe. Subfigure (c) covers aluminum pipes, showing 21% reduction with $90^\circ$ crack, 18.42% with $45^\circ$ crack, and 19.74% with $135^\circ$ crack against the healthy baseline.

**Fig. 14:**
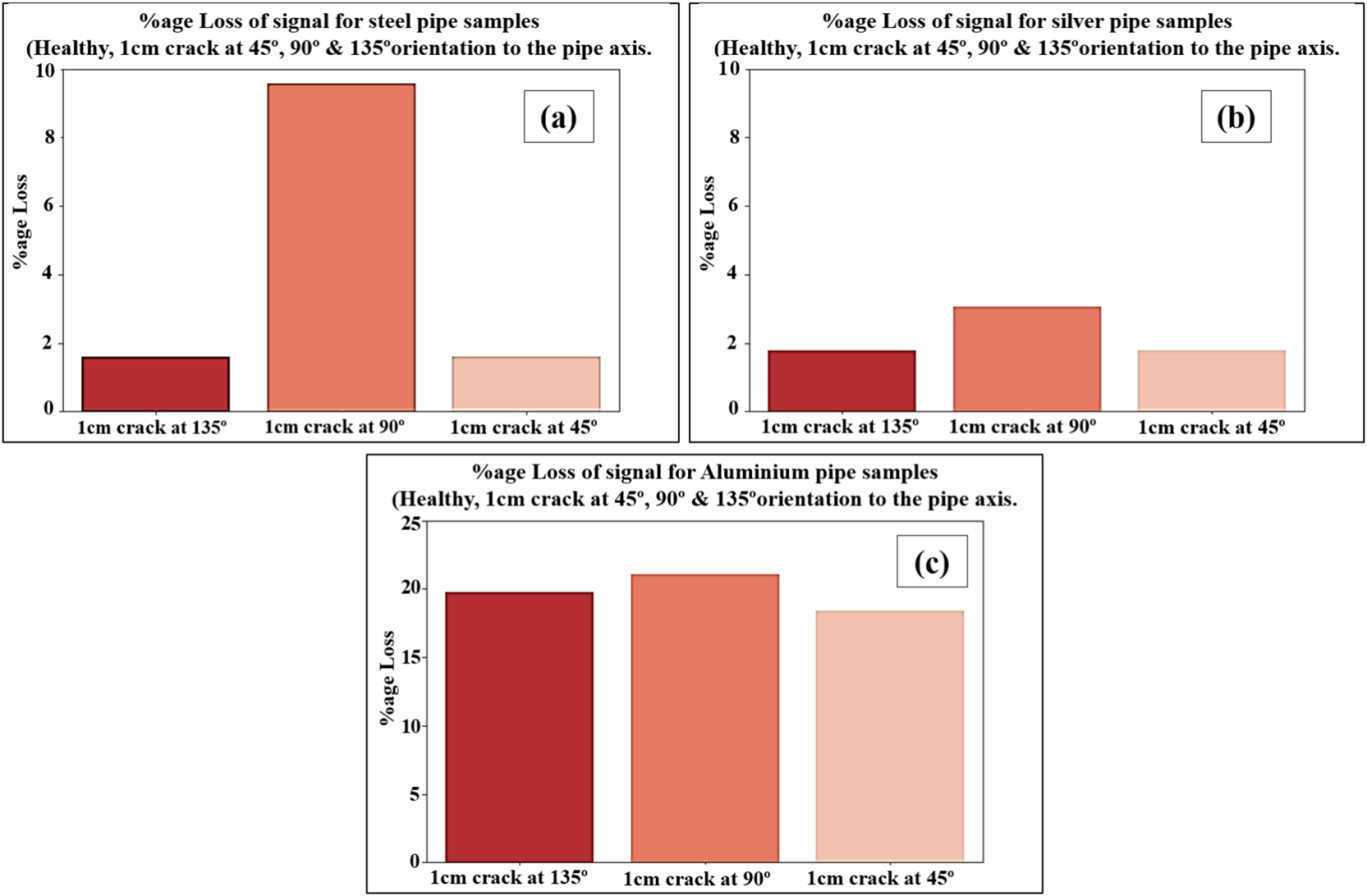
Percentage signal loss for 1 cm cracks at $135^\circ$, $90^\circ$, and $45^\circ$ (**a**) steel, (**b**) silver, and (**c**) aluminum pipes, compared with healthy pipe samples.

Figure 14 shows percentage signal loss for 1 cm cracks at $45^\circ$, $90^\circ$, and $135^\circ$ in steel, silver, and aluminum pipes compared to healthy samples. The bell-shaped signal loss pattern–rising from $0^\circ$ to $90^\circ$ and dropping from $90^\circ$ to $180^\circ$–comes straight from crack-beam geometry. At $90^\circ$, the ultrasonic beam slams the crack face head-on, triggering maximum backscattering and mode conversion. This tears apart the guided wave packet the worst, giving the highest energy loss. Tilt the crack to $45^\circ$ or $135^\circ$, and the beam hits at an angle, cutting down direct reflection and scattering. Some of the wave slides along or past the crack with less mess, so more energy comes back as a clean echo. This symmetry around $90^\circ$ builds the bell curve in all three materials, with the top loss at normal strike and the lowest near grazing angles.

### Crack localization & classification of defected pipelines samples

The experimental configuration used for crack localization in the tested pipes is described in the Fig. 11. A piezoelectric transducer (piezo probe) is mounted at one end of the specimen, while circumferential cracks of controlled depth are introduced at an unknown axial distance X from the transducer. By subtracting the healthy A-scan from each defective signal and detecting the significant reflected echo of the L(0,2) guided-wave mode, the time-of-flight (ToF) of the crack reflection is determined. Using the material-specific group velocity, the unknown distance X is calculated as shown in the Table 5. The crack localization accuracies of 97% for steel pipes, 90% for silver pipes, and 92% for aluminium alloy pipes were achieved, with maximum absolute errors across all materials.

**Table 5:** Crack localization. unknown X-distance from piezo prob & Accuracy for cracks prediction.

Sr No.	Sample	X-Distance (mm)	Accuracy
1	Steel pipe	118.5	97%
2	Silver pipe	83.5	90%
3	Aluminium pipe	107.1	92%

Following machine learning models, including XGBoost, CatBoost, LightGBM, Logistic Regression, and Random Forest, are implemented to classify defective and healthy pipes. Among these, Random Forest achieved the best performance in distinguishing healthy from defective pipes, exhibiting the highest accuracy, precision, recall, and F1-score across all tested materials as shown in the Table 6.

**Table 6:** Performance comparison of machine learning models for pipeline defect detection.

		Aluminium pipes	Silver pipes	Steel pipes
Sr No.	Model	F1-Score	F1-Score	F1-Score
1	Random Forest	0.8735	0.8505	0.8842
2	LightGBM	0.8358	0.7673	0.8761
3	XGBoost	0.8268	0.7548	0.8057
4	CatBoost	0.8233	0.7524	0.7959
5	Logistic Reg	0.7403	0.7334	0.7307

A comparative analysis of this work against previously reported ultrasonic-based NDT methods for pipelines is illustrated in the Table 7. According to this comparative analysis, the earliest studies relied on conventional signal-processing, which works on defect types, including corrosion, longitudinal cracks, butt-weld joints, and metal-loss features. A major contrast of all the studies to work on surface cracks using the pitch catch ultrasonic testing technique rather than pulse echo. A couple of studies present the implementation of AI and IoT for the automation of pipeline inspection, while robotics is integrated by a single source. In contracts, this work presents a novel solution and major contribution towards the inspection of pipelines by integrating robotics and AI. Pulse Echo ultrasonic testing technique is used to determine the effect of a circumferential crack on the surface of circular pipes. This work represents a significant improvement in both automation and generalization capability over prior ultrasonic-based non-destructive testing techniques.

**Table 7:** Comparative analysis of previous studies for the ultrasonic testing of pipeline inspection.

References	Year	Structure	L (mm)	OD (mm)	WT (mm)	Crack Type	LC	Sensor	MD	RM	AI
^39^	2021	Stainless Steel Pipe	–	114.3, 88.9, 73.025	–	Longitudinal Crack & Corrosion	S	Electromagnetic Acoustic Transducers	PC	$\checkmark$	–
^34^	2022	Steel Pipe	1000	112	1.9	Circumferential Cracks	S	PZT Strip	PC	$\times$	IoT
^28^	2022	Steel Pipe	1000	600	1	Through Hole (4mm $\phi$)	S	BSH-PT	PC	$\times$	-
^32^	2022	Steel Pipe	1000	610	10	Butt weld joint	S	PMUTs	PE	$\times$	–
^31^	2023	Steel Pipe	200	88	2	Corrosion	In	PZT	PC	$\times$	CNN
^29^	2023	PE* Pipe	–	200	20	Circumferential Cracks	S	Piezo Ceramics	PC	$\times$	–
^30^	2024	Steel Pipe	1000	114.6	4	Notch & Mass Loading	S	PZT	PC	$\times$	–
^38^	2025	Al Pipe	–	–	–	Bend + Blow out	S	PVDF	PC	$\times$	–
^33^	2025	Steel Pipe	–	–	–	Longitudinal Crack	S	UT sensor	PE	$\times$	–
This Work		Steel, Silver, & Al Pipe	270, 280, 300	20.5, 25.4, 27	varies	Circumferential Cracks	S	Piezo-Ultrasonic Transducer	PE	$\checkmark$	ML

Al Pipe:aluminium pipe; PE*:polyethylene pipe; L:nominal length of pipe; OD:outer diameter of pipe; WT:wall thickness of pipe; LC:location of cracks; S:surface cracks; In:internal cracks; BSH-PT:bidirectional SH wave piezoelectric transducer; PMUTs:piezoelectric micro ultrasonic transducers; MD:mode of SHM technique; PC:pitch-catch; PE:pulse-echo; RM:robotic manipulator.

## Conclusion

This study developed and validated an AI-enabled pulse-echo Structural Health Monitoring (SHM) system integrated with a robotic gripper for non-destructive testing of circular pipes. The system successfully captured longitudinal guided wave modes L(0,1) and L(0,2) and demonstrated reliable detection and localization of surface cracks across steel, silver, and aluminum pipes with varying geometries. Experimental results showed that pipe diameter, material properties, couplant usage, and crack orientation significantly influenced echo characteristics. Smaller diameters enhanced wave confinement and signal amplitude, while perpendicular cracks ($90^\circ$) caused the greatest signal loss. Crack localization was achieved using time-of-flight (ToF) analysis and material-specific group velocities. Machine learning models were employed to classify healthy and defective pipes, with the Random Forest classifier achieving the highest accuracy, precision, recall, and F1-score across all materials. The integration of a robotic manipulator enabled automated, precise, and repeatable inspections, highlighting the system’s suitability for industrial SHM applications. Despite these promising outcomes, the study was limited to controlled laboratory conditions and a restricted range of surface crack types. Environmental influences, complex defect morphologies, and additional guided wave modes were not considered Future work will focus on advanced signal processing and learning techniques, expanded datasets, additional wave modes, and more complex geometries and materials. Extending the system to real-world industrial environments with adaptive robotic inspection strategies will further enhance its robustness and applicability.

## Supplementary Information

Supplementary Information 1.

Supplementary Information 2.

Supplementary Information 3.

## Data Availability

All data supporting the findings of this study are available within the paper and its Supplementary Information.
